# Multiclass Classification of Hepatic Anomalies with Dielectric Properties: From Phantom Materials to Rat Hepatic Tissues

**DOI:** 10.3390/s20020530

**Published:** 2020-01-18

**Authors:** Tuba Yilmaz

**Affiliations:** Department of Electronics and Communication Engineering, Istanbul Technical University, Istanbul 34469, Turkey; tuba.yilmaz@itu.edu.tr; Tel.: +90-212-285-3561

**Keywords:** hepatic malignancies, in vivo dielectric properties, machine learning, k-nearest neighbors (kNN), logistic regression (LR), random forests (RF), liver phantoms

## Abstract

Open-ended coaxial probes can be used as tissue characterization devices. However, the technique suffers from a high error rate. To improve this technology, there is a need to decrease the measurement error which is reported to be more than 30% for an in vivo measurement setting. This work investigates the machine learning (ML) algorithms’ ability to decrease the measurement error of open-ended coaxial probe techniques to enable tissue characterization devices. To explore the potential of this technique as a tissue characterization device, performances of multiclass ML algorithms on collected in vivo rat hepatic tissue and phantom dielectric property data were evaluated. Phantoms were used for investigating the potential of proliferating the data set due to difficulty of in vivo data collection from tissues. The dielectric property measurements were collected from 16 rats with hepatic anomalies, 8 rats with healthy hepatic tissues, and in house phantoms. Three ML algorithms, k-nearest neighbors (kNN), logistic regression (LR), and random forests (RF) were used to classify the collected data. The best performance for the classification of hepatic tissues was obtained with 76% accuracy using the LR algorithm. The LR algorithm performed classification with over 98% accuracy within the phantom data and the model generalized to in vivo dielectric property data with 48% accuracy. These findings indicate first, linear models, such as logistic regression, perform better on dielectric property data sets. Second, ML models fitted to the data collected from phantom materials can partly generalize to in vivo dielectric property data due to the discrepancy between dielectric property variability.

## 1. Introduction

The open-ended coaxial probes have been widely used for broadband dielectric property characterization of high relative permittivity and high loss materials. The probes are mostly utilized for gathering dielectric property data in a laboratory setting to enable the development of microwave therapeutic and diagnostic applications. However, the current technique suffers from high measurement error rates. These error rates were reported to be as high as 30% for in vivo measurements. Such high error prevents the realization of the technique as a tissue characterization device [1].

The use of open-ended coaxial probes as a tissue characterization device was previously envisioned in the literature. In [2], the reported invention described insertion of an open-ended coaxial probe with a maximum aperture diameter of 0.79 mm into a needle biopsy guide for real-time diagnosis. In principle, such application would enable rapid diagnosis and cost minimization during biopsy. However, as pointed out in [2], cable movements in microwave systems cause calibration degradation introducing noise to measurements. This presented a challenge since the probe and the cable had to be moved in an in vivo setting. To minimize the effect of such error source, an electronic calibration module was proposed. Open-ended coaxial probes with larger apertures along with the electronic calibration kit are currently commercially available for laboratory use only. The reported measurement error is still as large as 5% for a laboratory setting [3].

In another study, a microwave tissue characterization system was presented [4]. The system proposes the ability to determine the anomaly of the material under test (MUT) with open-ended coaxial probes. The invention reported a vacuum mechanism located at the tip of the probe. This mechanism was intended to be used for MUT immobilization. Note that a loose contact between the probe tip and the MUT is known to contribute to high error rates. However, this invention only proposed a costly hardware update and it did not address other potential error sources.

In [5], a microwave pathology system was presented. The invention proposed to characterize the ex vivo dielectric properties and a microwave image of an excised tissue. It is known that several factors may contribute to the dielectric property discrepancy between in vivo and ex vivo tissues, such as tissue hydration, temperature, and blood perfusion [6]. Liver tissue is one of the widely studied samples in literature, where in [7], dielectric properties of human liver tissues were measured both in vivo and ex vivo. The reported results showed both a dielectric property discrepancy and a different dispersion behavior at higher frequencies between in vivo and ex vivo measurements. In [8,9], dielectric properties ex vivo and in vivo rat liver were reported. It was shown that the measured ex vivo relative permittivity was lower than the measured in vivo relative permittivity. In another study, to quantify the dielectric property change after excision, the dielectric properties of the mouse liver tissue were measured 5 min to 3.5 h after excision [10]. It was concluded that the relative permittivity was changed gradually up to 30%; similarly, conductivity changed over 35%. Therefore, the dielectric property change due to excision should be taken into account for the proposed system. While proposed pathological application of the technique in [5] describes a new application avenue, solutions to the aforementioned inherent error sources were not proposed.

As previously presented in the literature, the open-ended coaxial probes can be employed to realize novel diagnostic applications. However, research in this area has been stagnated due to inherent high measurement error of the technique. Although the research on qualitative imaging techniques might be considered as an alternative, these methods proposes to use an entirely different approach both mechanically and mathematically [11,12]. For instance, a microwave imaging system can not be used for real-time tissue characterization during surgeries in its current form. The microwave imaging systems ultimately aims to compete or aid the existing imaging methodologies including computed tomography (CT) and magnetic resonance imaging (MRI).

In [13], an open-ended coaxial probe fabricated with an RF cable was utilized to collect in vivo dielectric properties of malignant and healthy hepatic rat tissues. Collected dielectric properties were then used to train and test a binary SVM algorithm. The algorithm was able to classify the hepatic malignancies with 98.3% accuracy. The reported study in [13] indicates that it is possible to perform high accuracy classification of the tissue via evaluating the single measurement response of the open-ended coaxial probe measurements with machine learning (ML) algorithms. However, in general, tissues are heterogeneous and anomalies can have different stages. For example, in [13] cirrhosis tissues were observed along with hepatic malignancies. The dielectric properties of cirrhosis tissues have a high overlap with malignant tissues. Therefore, there is a need to utilize multiclass classification algorithms to evaluate the realistic performance of the machine learning (ML) algorithms. Additionally, in vivo measurements are cumbersome, costly, requires staff and facilities. Therefore, there is a need to study data proliferation methods.

The goals of this study are two folds: (1) To investigate the effectiveness of ML algorithms on the classification of healthy, cirrhosis, and malignant in vivo dielectric property data, and (2) to investigate the potential data proliferation techniques with phantom materials. The contributions are further explained below,

- Application of multiclass ML algorithms to in vivo dielectric property data: Performed by applying multiclass ML algorithms to in vivo rat dielectric properties collected from rat hepatic tissues including healthy, cirrhosis, and malignant tissues. This approach reveals the ability of the technique to discriminate different pathological stages of a diseased tissue in a realistic scenario.
- Potential proliferation of the data with phantoms: In vivo data collection is laborious, costly, requires facilities, and subject to strict ethical regulations. On the other hand, ML algorithms thrives with large amount of data. Therefore, there is a need to proliferate the data. One option is to acquire the data from phantom materials. This was performed by first, collecting dielectric property data from phantom materials mimicking the dielectric properties of liver tissues. Next, classifying the collected dielectric property data with multiclass ML algorithms. Finally, generalizing the model to in vivo dielectric property measurements.

The dielectric properties of each tissue group and phantoms, uncertainty analysis, statistical analysis along with the performance of classification algorithms are given in this work. The remainder of this paper is organized as follows: Section 2 describes the methodology used for dielectric property measurements, data analysis, and ML algorithms. Results are given in Section 3, a discussion is given in Section 4 and lastly, the conclusions are drawn in Section 5.

## 2. Materials and Methods

This section details the methodology followed for in vivo, phantom, and system uncertainty experiments. Additionally, a brief description of the ML algorithms are provided in this section.

### 2.1. In Vivo Dielectric Property Measurements

In vivo dielectric property measurement including preparation of measurement samples, the measurement setup, and methodology followed during measurements are explained in this section.

#### 2.1.1. Experiment Samples

A total of 30 adult female Wistar albino rats (120 days old) were obtained from the Institute of Experimental Medicine and Research at Istanbul University. Six animals in the control group received an intraperitoneal 0.1 M NaCl solution once a week for 10 weeks. The experiment group received an intraperitoneal injection of 50 mg/kg Diethylnitrosamine (Sigma Chemical Company, St. Louis, MO, USA) saline solution once a week for 10 weeks. The animals were anesthetized with an intraperitoneal injection of 80 mg/kg ketamine + 10 mg/kg xylazine mixture starting from week 16. In vivo dielectric property measurements were taken every 2 weeks. The animals were sacrificed immediately after the completion of dielectric property measurements.

#### 2.1.2. Measurement Setup

**Figure 1:**
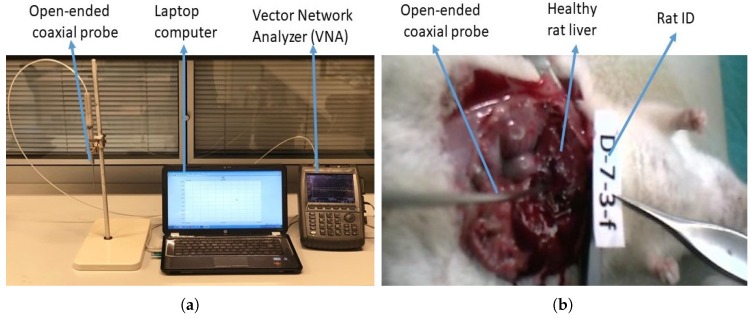
Dielectric property measurement setup: (**a**) measurement setup in a laboratory environment with Agilent Fieldfox N9923A Network Analyzer (NA), laptop computer, and integrated probe; (**b**) a picture taken during in vivo dielectric property measurements displaying the exposed liver of the experiment animal and the open-ended coaxial probe.

The dielectric property measurements were performed using an open-ended coaxial probe with a 2.2 mm aperture produced by Mitos Medical Technologies described in [13]. The probe was integrated into an RF coaxial cable to eliminate the problems that may arise from microwave cable connections. The measurements were collected with Agilent Fieldfox N9923A Network Analyzer (NA) along with 85070E software between 500 MHz to 6 GHz with 500 MHz intervals. This allowed sampling of the dielectric properties at enough frequency points for classification purposes. A picture of the measurement setup displaying the laboratory setting and a picture from the in vivo animal experiments are given in Figure 1a,b, respectively.

#### 2.1.3. In Vivo Measurements

Standard open, short, known material calibration steps were performed before measurement. That is, the probe aperture was ended with air, conductive textile, and deionized water. Measurements collected immediately after the excision were named wet measurements. After collection of wet measurements, the location of the measurement was wiped with a 0.1 M NaCl solution and the second set of measurements were collected, which were called dry measurements. This procedure was followed because the 0.1 M NaCl solution is widely used during surgical operations. During cancer resection surgeries, the surgical area is washed and wiped to clear the accumulated blood or subcutaneous fluid. Further explanation is provided on Section 4. In this work, whole data sets were used for training the ML algorithms. Therefore, relevant data analysis was performed on combined data set.

### 2.2. Phantom Characterization and Measurements

Phantom materials have been widely utilized in the literature to test microwave diagnostic technologies. The animal experiments are costly, time consuming, and it must satisfy several ethical requirements. Therefore, data collection from animals is cumbersome. On the other hand, the ML algorithms are known to perform better with large data sets. To increase the data set, an option is to consider collecting data from phantom materials to proliferate the training data. This method allows: (1) validation of the models with another dielectric property data set, (2) explores the potential for the generalization of the model trained on phantom dielectric property data to the in vivo data set. The methodology followed for phantom characterization and dielectric property collection are given below.

#### 2.2.1. Phantom Materials

The phantoms were characterized by varying the oil amount in the oil-in-gelatine dispersion phantom recipes given in [14]. The recipe is used due to the simplicity and proven ability to represent the biological tissue dielectric properties for wide band applications. A base recipe is formed by using 230 g deionized water, 34.1 g gelatine, 40 g surfactant (dish-washing liquid), and 1.2 g NaCl. Then, to form the malignant, cirrhosis, and healthy tissue mimicking materials 25, 40, and 55 g of sunflower oil were used, respectively. The phantoms were formed by first, melting the gelatine in 100 g deionized water. Next, when the mixture was cooled to 35 ${}^{\circ }$C, remaining deionized water, surfactant, and NaCl was added while stirring slowly. Then, at 28 ${}^{\circ }$C, the oil was added to the mixture while stirring slowly. Dielectric properties were collected after the phantoms were left to solidify for 48 h.

#### 2.2.2. Measurement Setup

**Figure 2:**
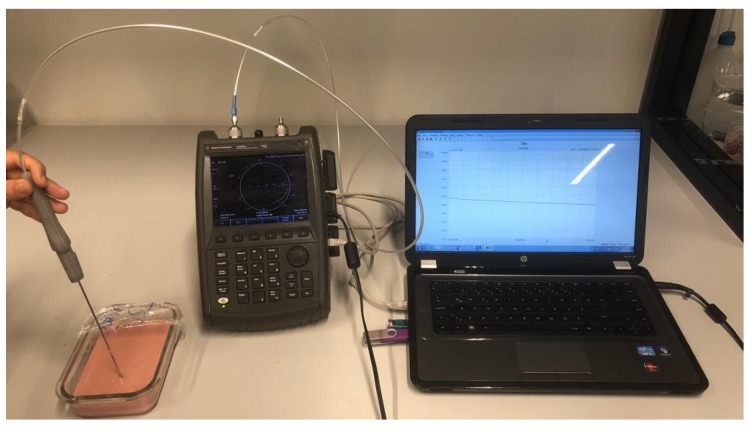
Phantom material and measurement setup.

Agilent Fieldfox N9923A NA along with 85070E software, were used during phantom measurements. Calibration was performed using a standard open, short, deionized water calibration procedure. The measurements were collected from different parts of the phantoms including top, bottom, and sides. Five measurements were collected from each point. The phantom temperatures were 21.9 ± 0.2 ${}^{\circ }$ C. A total of 90 measurements were collected from each phantom material. Measurement set up and a phantom sample is shown in Figure 2.

### 2.3. Uncertainty Analysis

The uncertainty calculation described in [15,16] was adopted in literature for dielectric property measurements of biological tissues to identify the possible error sources [17]. Factors contributing to the uncertainty were listed as measurement repeatability, systematic errors, system drift, cable movements [17,18].

In this work, to quantify the system uncertainty, dielectric properties of six 0.1 M NaCl solutions were measured. The solutions were prepared by mixing NaCl with deionized water. Four uncertainty sources listed below were analyzed:

- Repeatability: Dielectric property measurements of 0.1 M NaCl solution were collected at different sessions. To calculate uncertainty due to repeatability, standard deviation from the mean (SDM) was calculated from a total of 54 measurements.
- Systematic errors: It was calculated by taking the difference between the measured dielectric properties; that is, mean of the 54 measurements collected for SDM calculations, and reported literature data [19,20].
- System drift: It was calculated by taking the difference between the dielectric property measurement after calibration and dielectric property measurement 30 min after calibration. Thirty min was chosen since during in vivo rat liver measurements, the system was re-calibrated every thirty minutes. To calculate the system drift, 30 measurements were used.
- Cable movements: It was characterized by calculating the difference between measured dielectric properties of 0.1 M NaCl solutions for different cable positions. Note that the most extreme cable movements that could occur during in vivo measurements were considered. A total of 68 measurements were used to calculate the uncertainty due to cable movements.

All measurements were collected at 25 ${}^{\circ }$C. This temperature was chosen since, during in vivo measurements, the temperature of the sample could range from 25 ${}^{\circ }$C to 37 ${}^{\circ }$C. Standard uncertainties were first calculated for each frequency point. Next, the mean of the all calculated uncertainties were taken to characterize the individual uncertainty at the frequency range of interest. Calculation results are given in Section 3.1.

### 2.4. Multiclass Classification

Multiclass classification algorithms were used to categorize the rat hepatic tissue and the phantom dielectric properties in a reproducible manner. The supervised models fitted to the phantom data set are then generalized to the rat hepatic tissue data set. Among other supervised learning algorithms, k-nearest neighbors (kNN), logistic regression (LR), and random forest (RF) algorithms were used to perform the classification. The chosen algorithms represents three groups of methods namely, instance-based, linear, and ensemble methods. Instance-based learners stores the training data in the memory instead of constructing a model and compares the new test data to closest saved instances to perform the prediction. The linear methods are known to construct a simple model that is, in some instances, known to generalize well due to simplicity. Ensemble methods combine weak classifiers and determines the class with a voting mechanism. This way, the ensemble methods forms a strong classifier from weak classifiers. These three benchmark methods are compared to understand the performance of each method on the collected wide band dielectric property data which involves correlated features. The algorithms are briefly explained in the following section.

#### 2.4.1. k-Nearest Neighbors (kNN)

kNN is an instance-based lazy learner. The algorithm is inherently multiclass and have been utilized for a variety of applications, from text categorization to classification of renal calculi [21,22]. During training, the algorithm stores the training data to compare them with a newly introduced data. The comparison is made by calculating the distance between the training and test data to find the k-nearest neighbors. The distance can be calculated with different methods including mostly used Euclidean distance. Both the number of neighbors and distance calculation method initialized by the user can be optimized based on the metric evaluations. The algorithm is easy to implement and optimize; because, it does not require tuning several parameters. Since it does not build a model, the algorithm spends less time during training and more time during the prediction. The expected disadvantage could be the speed and memory requirements as the data in training set is increased.

#### 2.4.2. Logistic Regression (LR)

LR is mostly used to perform binary classification for many different applications including the financial and healthcare areas. The algorithm also forms one of the building blocks of deep learning. LR works by forming a linear model that would encompass the relationship between the features and labels. Based on the linear model, class probability of a test sample is calculated which is then converted to a binary value for class assessment. The binary classification can easily be expanded to a multiclass categorization by adopting one-vs-one or one-vs-rest schemes.

#### 2.4.3. Random Forest (RF)

RF is a non-linear classifier and works by forming non-linear boundaries via combining linear boundaries. RF algorithm is also an ensemble method that combines weak decision tree classifiers [23]. The goal of the decision tree is to obtain a final node with a data that belongs to a single class; namely, a pure leaf node. This is done by creating a set of rules that iteratively divide the sample data set until the leaf node is reached. In the RF algorithm, the decision trees are allowed to grow without pruning on the subset of the data. Then, the class of a sample is decided with majority voting. In this way, each decision tree learn different representations of data. These independent weak decision tree classifiers provide a generalized model. The RF algorithm is inherently a multiclass classifier and it is a powerful ensemble method that can prove to be a good candidate for dielectric property based classification of liver tissues [24].

## 3. Results

### 3.1. Uncertainty Calculation with 0.1 M NaCl Solution

The method performed during the uncertainty analysis are given in Section 2.3. The individual contribution of each uncertainty source, as well as the combined uncertainty, is given in Table 1.

The combined uncertainty for relative permittivity agrees with the literature data given in [18]. Combined uncertainty for relative permittivity and conductivity is slightly higher than the calculated values given in [17]. Nevertheless, the uncertainty trends (e.g., the system uncertainty is higher for conductivity) are consistent with the literature data.

### 3.2. Dielectric Properties of Healthy and Diseased Rat Liver Tissues

**Figure 3:**
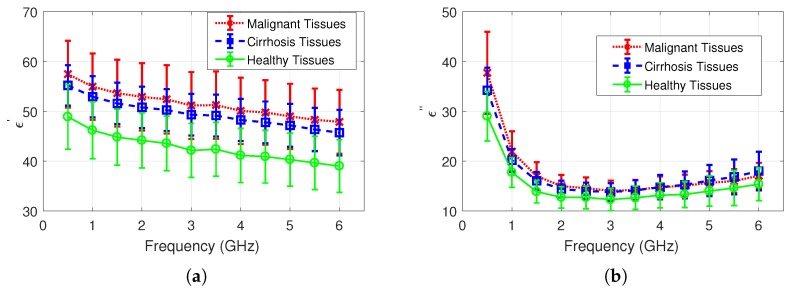
Measured dielectric properties of healthy and diseased rat liver tissues: (**a**) relative permittivity comparison of malignant, cirrhosis, and healthy rat liver; (**b**) dielectric loss comparison of malignant, cirrhosis, and healthy rat liver.

Comparisons of measurements collected from malignant, cirrhosis, healthy tissues are shown in Figure 3a,b for relative permittivity and dielectric loss, respectively. Note that each measurement group consists of 95 measurement samples. Mean relative permittivity and dielectric loss discrepancy between cirrhosis and malignant tissues are 4% and 1.8%, respectively. Mean relative permittivity and dielectric loss discrepancy between cirrhosis and healthy tissues are 16% and 14%, respectively. Lastly, the mean relative permittivity and dielectric loss discrepancy between malignant and healthy tissues are 21% and 16%, respectively. All discrepancies were calculated for the measurement frequency band from 0.5 to 6 GHz. Mean dielectric properties for malignant, cirrhosis, and healthy tissues and the calculated combined uncertainty values are given for three frequencies in Table 2.

### 3.3. Statistical Analysis

**Figure 4:**
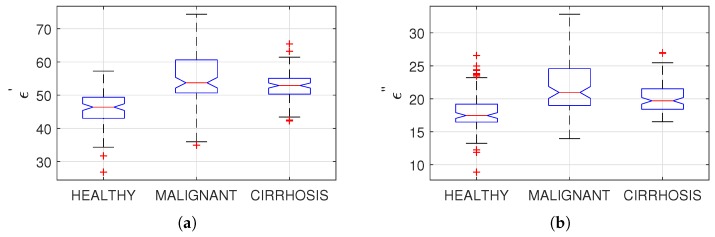
Comparison of mean, median, and standard deviation of malignant, cirrhosis, and healthy rat liver tissues at 1 GHz: (**a**) for relative permittivity; (**b**) for dielectric loss.

The statistical analysis was performed for each measured frequency. The sources of variability were analyzed by applying a one-way nested analysis of variance (ANOVA) to relative permittivity and dielectric loss values separately. The tests were performed at a 0.05 level of significance (α) and the obtained probability values (*p*-value) were smaller than the level of significance for both relative permittivity and conductivity. This result shows that there are statistically significant differences between the diseased and healthy rat liver tissues. The mean and standard deviation at 1 GHz are shown in Figure 4a,b for relative permittivity and dielectric loss, respectively. The mean dielectric properties for each group and *p*-values are given at three different frequencies in Table 3.

### 3.4. Dielectric Properties of Tissue Mimicking Materials

**Figure 5:**
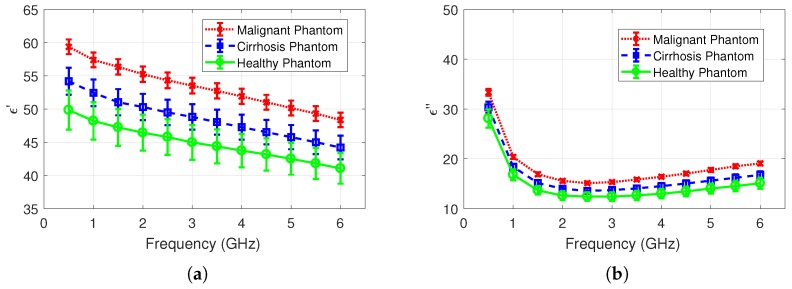
Measured dielectric properties of phantoms: (**a**) relative permittivity comparison of malignant, cirrhosis, and healthy phantoms; (**b**) dielectric loss comparison of malignant, cirrhosis, and healthy phantoms.

Measured permittivity and dielectric loss of the three phantom materials are given in Figure 5a,b, respectively. Mean permittivity discrepancy between phantom and in vivo measurements are 13.7%, 1.98%, and 24.5% for malignant, cirrhosis, and healthy tissues in the whole frequency band, respectively. Similarly, mean dielectric loss discrepancy between phantom and in vivo measurements are 15.7%, 4.09%, and 21.7% for malignant, cirrhosis, and healthy tissues in the whole frequency band, respectively.

Ultimately, the measurements were performed trying to imitate the in vivo measurement conditions. However, the in vivo conditions also includes the tissue heterogeneity and body fluids in the measurement site. Therefore, the phantoms can not fully represent the range of the in vivo measurements. This can further be simulated by characterizing phantoms with varying ingredients.

### 3.5. Multiclass Classification

**Figure 6:**
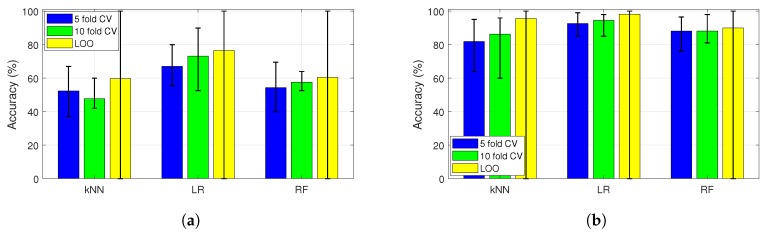
Dependence of Machine learning (ML) algorithms’ accuracies to cross validation (CV) scheme: (**a**) application of k-nearest neighbors (kNN), logistic regression (LR), and random forest (RF) algorithms by using 5-fold CV, 10-fold CV, leave one out (LOO) schemes to in vivo dielectric properties; (**b**) application of kNN, LR, and RF algorithms by using 5-fold CV, 10-fold CV, LOO schemes to phantom dielectric properties.

The multiclass classification is performed by using the dielectric property data sets collected from both the in vivo and phantom experiments. The in vivo dielectric property data contained 95 samples from each tissue category and the phantom dielectric property data included 90 samples from each phantom type. Due to the relatively small number of data, ML algorithm accuracies were evaluated by using different cross validation (CV) schemes including 5-fold CV, 10-fold CV, and leave one out (LOO) CV. The k-fold cross validation and LOO schemes are inherently capable of composing unique data sets since each fold includes a number of randomly selected samples from the original data set. Obtained accuracy values are shown in Figure 6a,b for in vivo and phantom dielectric property data sets, respectively. It can be seen from Figure 6 the algorithms shows discrepancy based on the chosen CV scheme. Since the number of data was limited when compared to conventional ML data sets LOO results can be considered more reliable; thus, LOO CV scheme was used for the next phase of evaluations.

**Figure 7:**
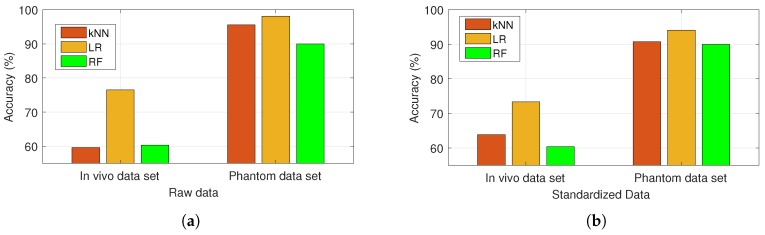
Comparison of k-nearest neighbors (kNN), logistic regression (LR), and random forest (RF) algorithm accuracies with leave one out (LOO) scheme: (**a**) application to raw data obtained from in vivo and phantom dielectric property measurements; (**b**) application to standardized data obtained from in vivo and phantom dielectric property measurements.

Comparison of ML algorithm accuracy values obtained using LOO scheme are shown in Figure 7a,b for raw and standardized data sets, respectively. The raw data consisted of 12 permittivity and 12 dielectric loss values from 0.5 to 6 GHz, represented as 24 features. The standardized values were obtained with z=(x−u)/s; where, *x* is the feature, *u* is the mean of the training samples of the feature, and *s* is the standard deviation of the training samples of the feature. Ultimately, the in vivo data was noisy and the cirrhosis tissue dielectric properties were very close to malignant ones; thus, the performance of ML algorithms were lower when compared to those obtained for phantom data sets. However, consistently the LR algorithm performed better on both data sets as well as the standardized data sets. The accuracy values obtained using the LR algorithm were 76.49%, 73.33%, 98.14%, and 94.07% for raw, standardized in vivo and phantom dielectric property data sets, respectively. This indicates that LR as a linear model generalizes well on dielectric property data. It should also be noted that the features namely, wide band permittivity and dielectric loss values are positively correlated; therefore, the linear model might allow to minimize the effect of the irrelevant features which in turn helps the ability of the model to generalize.

The models fitted to phantom data was also tested with in vivo dielectric property data. The best results were obtained by using the standardized data with slight difference between LR and kNN models. Obtained results are given in Table 4. Due to the mismatches on the mean and standard deviation of phantom and in vivo dielectric property data, the test accuracies drastically reduced to 48% and 50% for LR and kNN models, respectively. Despite this drop, we can state that further improvement can be done by expanding the phantom dielectric property data set by simulating the sample heterogeneity.

## 4. Discussion

To compare the collected in vivo dielectric property data with literature, a further analysis was performed by calculating the discrepancy between the measured mean in vivo dielectric property data and previously reported measurements. The literature data includes healthy and diseased liver tissue dielectric properties collected from different species. The comparisons are given in Table 5. Note that the percent difference for the whole frequency band was calculated with Equation (1).

(1) $$\%Ɗ\varepsilon =\frac{1}{N}\sum_{i=1}^{N}\left|\frac{\varepsilon_{i}-\bar{\varepsilon_{i}}}{\bar{\varepsilon_{i}}}\right|*100$$

where *N* is the number of frequency points (*N* = 12), $\varepsilon_{i}$ and $\bar{\varepsilon_{i}}$ are the measured and literature dielectric properties, respectively.

It can be seen from Table 5 that the dielectric property discrepancy can vary based on the measurement conditions. As it was previously explained in the methodology section, the measurement site was wiped with 0.1 M NaCl solution to clean the accumulated blood and other body fluids in the measurement area. Two questions repeatedly raised regarding the use of 0.1 M NaCl solution: why it was used and whether it effects the tissue conductivity. During surgical resection, the physicians frequently use 0.1 M NaCl solution to protect tissue from drying and to clean the fluids in the area. Therefore, merely a standard realistic procedure during the in vivo experiments was applied in this work. To further analyze the effect of this on the conductivity of the tissue, a quantitative analysis between wet and dry measurements was carried out. The discrepancy for relative permittivity between wet and dry healthy measurements was 3.01 units at 1 GHz. Discrepancy between dry relative permittivity measurements of malign and cirrhosis tissues were not as significant, 1.3 units and 0.1 units at 1 GHz, respectively. A very small decrease in dry conductivity measurements were observed for all liver tissue types (up to 0.2 S/m at 1 GHz). Since conductivity did not increase for dry measurements, accumulation of the blood in the measurement site during wet measurements should be the source of discrepancy for dielectric properties. Further, the conductivity of the 0.1 M NaCl solution is close to body tissues. When compared at 1 GHz 1.22 (S/m) for 0.1 M NaCl solution [20], 0.90 (S/m) for healthy liver tissue [26], and 1.58 (S/m) for blood [26]. Therefore, temporary exposure of the liver tissues to 0.1 M NaCl does not to alter the conductivity of the tissue.

To expand the analysis, the models fitted to the in vivo raw dielectric property data were used for testing of the literature data, given in Table 6. A maximum 50% accuracy was obtained with the LR algorithm. One important point that needs to be noted is that the dielectric properties are effected from other factors such as the temperature and the hydration of the sample. For example, cool temperatures increases the dielectric properties and it is known that the ex vivo tissue temperatures are lower than in vivo counterparts. Therefore, representing the dielectric property collection conditions as a feature can increase the accuracy of the models which can potentially generalize well to other data sets.

In this work we have shown that the accuracy of the practical in vivo measurements can be increased to 76%, which is 6% higher than the dielectric property measurement accuracy rates given in literature. Similarly, over 98% accuracy is obtained with the phantom dielectric property data that is collected under relatively crude measurement conditions. These results are expected to improve with the expansion on the data base. However, in vivo measurements are cumbersome and costly to collect. Therefore, if one can increase the training data set by using phantoms that can increase the 76% accuracy obtained for in vivo measurements, this approach can potentially help the next phase of evaluations.

## 5. Conclusions

An open-ended coaxial probe measurement technique was utilized for measuring the dielectric properties of biological tissues. The technique is known to be error-prone; however, it is preferred due to its broadband measurement capabilities and flexible sample size requirements. Such advantages are making the technique attractive for tissue characterization. To employ the measurement technique as a tissue characterization device, accurate single measurements need to be collected from the tissue samples. To do so, three ML algorithms were trained with the collected in vivo complex permittivity measurements from malignant, cirrhosis, and healthy rat hepatic tissues. A total of 285 measurements with 95 samples from each tissue group were used. A 76% accuracy score was achieved with the LR algorithm. The ML models were also fitted to dielectric properties collected from phantom materials which was able to classify the data with over 98% accuracy with LR algorithm. The models were generalized and tested with in vivo measurement and literature data which resulted in a maximum 48% and 50% accuracies with LR algorithm, respectively. The LR algorithm resulted in better accuracy values and has the potential to generalize better comparing to kNN and RF models for the dielectric property data set. Finally, the obtained results indicate that the ML algorithms can be used for obtaining higher accuracies than the reported system accuracies in the literature.

## Figures and Tables

**Table 1:** Calculated uncertainty values for 0.1 M NaCl solutions between 0.5 and 6 GHz.

Dielectric Properties	Repeatability	Difference from the Literature [19,20]	System Drift	Cable Movement	Combined Uncertainty	Expanded Uncertainty
$\varepsilon^{{}^{\prime }}$	0.37	0.58	0.53	0.33	0.93	1.86
$\varepsilon^{{}^{\prime \prime }}$	1.27	3.17	2.19	0.58	4.09	8.18
σ(S/m)	0.15	0.36	0.23	0.10	0.47	0.94

**Table 2:** Comparison of mean measured in vivo dielectric properties of rat hepatic tissues along with combined uncertainty values.

Frequency (GHz)	Dielectric Properties	Malignant	Cirrhosis	Healthy
1.0	$\varepsilon_{r}$	54.97 ± 6.71	52.95 ± 4.23	46.20 ± 5.79
	$\varepsilon^{{}^{\prime \prime }}$	21.89 ± 5.65	20.19 ± 4.51	17.74 ± 4.93
	σ(S/m)	1.22 ± 0.49	1.12 ± 0.46	0.99 ± 0.47
3.0	$\varepsilon_{r}$	51.18 ± 6.78	49.33 ± 4.30	42.15 ± 5.49
	$\varepsilon^{{}^{\prime \prime }}$	14.06 ± 4.39	13.80 ± 4.29	12.29 ± 4.50
	σ(S/m)	2.35 ± 0.55	2.30 ± 0.53	2.05 ± 0.58
5.0	$\varepsilon_{r}$	48.99 ± 6.61	47.19 ± 4.40	40.31 ± 5.42
	$\varepsilon^{{}^{\prime \prime }}$	15.66 ± 4.53	16.11 ± 5.00	13.99 ± 4.94
	σ(S/m)	4.36 ± 0.78	4.48 ± 0.98	3.89 ± 0.95

**Table 3:** Mean dielectric properties and *p*-values of selected measurements at three different frequencies.

Frequency (GHz)	Dielectric Properties	Malignant	Cirrhosis	Healthy	*p*-Value
2.0	$\varepsilon^{{}^{\prime }}$	44.33	53.41	50.81	<0.01
	$\varepsilon^{{}^{\prime \prime }}$	12.88	15.12	14.39	<0.01
4.0	$\varepsilon^{{}^{\prime }}$	41.26	50.65	48.27	<0.01
	$\varepsilon^{{}^{\prime \prime }}$	13.27	14.98	14.77	<0.01
6.0	$\varepsilon^{{}^{\prime }}$	38.98	48.38	45.72	<0.01
	$\varepsilon^{{}^{\prime \prime }}$	45.72	17.40	18.01	<0.01

**Table 4:** Metric scores obtained by using the models fitted to standardized phantom data on testing the in vivo dielectric property data.

Model	Accuracy (%)	Precision (%)	Recall (%)	F1 score (%)
LR	48	48	48	47
kNN	50	54	50	50

**Table 5:** Comparison of measured mean in vivo dielectric properties of malignant, cirrhosis, and healthy tissues with literature data.

Tissue Type	$\Delta \varepsilon^{\prime }\left(\%\right)$	$\Delta \varepsilon^{\prime \prime }\left(\%\right)$	Reference	Species	Condition
Malignant	3.64	53.91	O’Rourke et al. [7]	human	ex vivo
	18.17	13.31	Peyman et al. [25]	human	ex vivo
Cirrhosis	2.14	13.81	O’Rourke et al. [7]	human	ex vivo
	14.8	19.12	Peyman et al. [25]	human	ex vivo
Healthy	1.58	3.85	Gabriel et al. [26]	human	N/A
	4.66	35.12	O’Rourke et al. [7]	human	ex vivo
	9.10	10.29	Lazebnik et al. [27]	human	ex vivo
	4.13	13.81	Abdilla et al. [28]	porcine	ex vivo

**Table 6:** Metric scores obtained by using the models fitted to in vivo raw dielectric property data on testing of the literature data.

Model	Accuracy (%)	Precision (%)	Recall (%)	F1 Score (%)
LR	50	50	50	46
kNN	44	53	44	47
